# Distinguishing intentional from nonintentional actions through eeg and kinematic markers

**DOI:** 10.1038/s41598-023-34604-y

**Published:** 2023-05-25

**Authors:** C. C. Derchi, E. Mikulan, A. Mazza, S. Casarotto, A. Comanducci, M. Fecchio, J. Navarro, G. Devalle, M. Massimini, C. Sinigaglia

**Affiliations:** 1 Present Address: IRCCS, Fondazione Don Carlo Gnocchi ONLUS, 20148 Milan, Italy; 2 Department of Health Sciences, Università Degli Studi di Milano, Via di Rudinì 8, 20146 Milan, Italy; 3 Department of Biomedical and Clinical Sciences, Università Degli Studi Di Milano, Via G. B. Grassi 75, 20157 Milan, Italy; 4 Center for Neurotechnology and Neurorecovery, Department of Neurology, Massachusetts General Hospital, Boston, MA USA; 5 Department of Philosophy, Università Degli Studi Di Milano, Via Festa del Perdono 7, 20122 Milan, Italy; 6 Cognition in Action (CIA) Unit, PHILAB, 20122 Milan, Italy; 7 Department of Philosophy, Stanford University, Stanford, CA USA

**Keywords:** Neuroscience, Psychology

## Abstract

How can an intentional movement be distinguished from the same movement done nonintentionally? How can this distinction be drawn without asking the subject, or in patients who are unable to communicate? Here we address these questions, by focusing on blinking. This is one of the most frequent spontaneous actions in our daily life, but it can also be done intentionally. Furthermore, blinking is often spared in patients with severe brain injuries, and for some, it is the only way to report complex meanings. Using kinematic and EEG-based measures, we found that intentional and spontaneous blinking are preceded by different brain activities, even when they are indistinguishable. Unlike spontaneous ones, intentional blinks are characterized by a slow negative EEG drift, resembling the classic readiness potential. We investigated the theoretical implication of this finding in stochastic decision models as well as the practical significance of using brain-based signals to improve the discrimination between intentional and nonintentional actions. As proof of principle, we considered three brain-injured patients with rare neurological syndromes characterized by motor and communicative impairments. Although further research is needed, our results indicate that brain-based signals can offer a feasible way to infer intentionality even in absence of overt communication.

## Introduction

Some actions can be either intentional or nonintentional despite being externally indistinguishable. For example, we might intentionally blink or cough to attract another’s attention even though the same actions typically occur nonintentionally. How can nonintentional events be distinguished from things done intentionally? How can this distinction be drawn without asking the subject or in patients who cannot communicate because of severe motor and cognitive disabilities? Here, we exploit eye blinking as an almost ideal model to explore the boundary between intentional and nonintentional actions.

A blink consists of rapid eyelid closure and opening mediated by the reciprocal action of two main antagonistic muscles, the *orbicularis oculi* and *levator palpebrae superioris*, respectively^1–4^. While intentional blinks can convey complex meanings, spontaneous blinks are one of the most frequent nonintentional actions we make in our daily lives. Unlike reflexes or other externally driven movements typically considered nonintentional^5^, spontaneous blinks can be generated internally and occur regularly without needing external triggers^6^. Finally, brain-injured patients suffering from severe motor and cognitive disorders may only be able to communicate through blinks^7–9^, as they may be spared even in these conditions. Therefore, distinguishing between spontaneous and intentional blinking can be valuable for assessing a patient's ability to act intentionally.

The distinction between intentional and nonintentional blinking has been previously studied by appealing to either kinematic or electroencephalographic (EEG) markers. Indeed, intentional and nonintentional eye blinks have been characterized by distinct waveforms of the electrooculogram (EOG) and the electromyogram (EMG), with the intentional blinking exhibiting a significantly greater amplitude as compared to the spontaneous eye blinks^10–12^. Some studies used eye blinks within brain-computer interfaces (BCI) systems, combining the modulation of EOG amplitude and the amplitude of spontaneous and intentional eye blink artifacts on the EEG signal^13,14^. As for the EEG marker, a restricted number of studies have found intentional blinking to be prefaced by a negative slow brain wave, resembling the *Bereitschaftspotential* or Readiness Potential (RP)^15^. At the same time, this was not the case for spontaneous blinking^16,17^.

However, previous attempts did not investigate the key issue of the relationship between the kinematic and EEG markers. Does a purely brain-based EEG marker provide additional information above and beyond kinematic markers? Further, can the EEG marker differ between intentional and nonintentional blinks even when the corresponding kinematic features are comparable? These questions are relevant for elucidating the neuronal substrates of intentional and nonintentional actions and for developing practical ways of inferring intentionality from electrophysiological signals in the context of patients’ stratification for tailored treatment and rehabilitation.

To address these questions we recorded EOG and EEG activity from healthy participants when they blinked either spontaneously or intentionally. Participants were also asked to perform intentional eye blinks either quickly or slowly to dissociate kinematics (as recorded by the EOG) and intentionality (as recorded by the EEG). In this way, we could analyze cases where intentional and nonintentional blinks had similar kinematics but differed in intentionality, as well as cases where the opposite was true. We hypothesized that if the brain-based (EEG) marker carries critical information to distinguish intentional from nonintentional blinks, it should discriminate the two kinds of blinks when their kinematics (EOG) was similar. Conversely, we hypothesized that the intentional blinks that varied in kinematics, depending on whether they are fast or slow, should share the EEG correlate.

To further investigate the contribution of the EEG marker to the discrimination between intentional and nonintentional actions, we run a series of regression analyses on data from healthy participants. As proof of principle, we then applied these models to data from three brain-injured patients with rare neurological syndromes characterized by severe motor and communicative impairments. While our models are still in the early stages of validation, we are confident that they present a valuable approach for distinguishing intentional from nonintentional actions in brain-injured patients. In cases where such distinctions are particularly challenging or even impossible to make, this tool could prove especially promising.

## Results

### EEG but not the EOG markers distinguished intentional from spontaneous blinking

**Figure 1:**
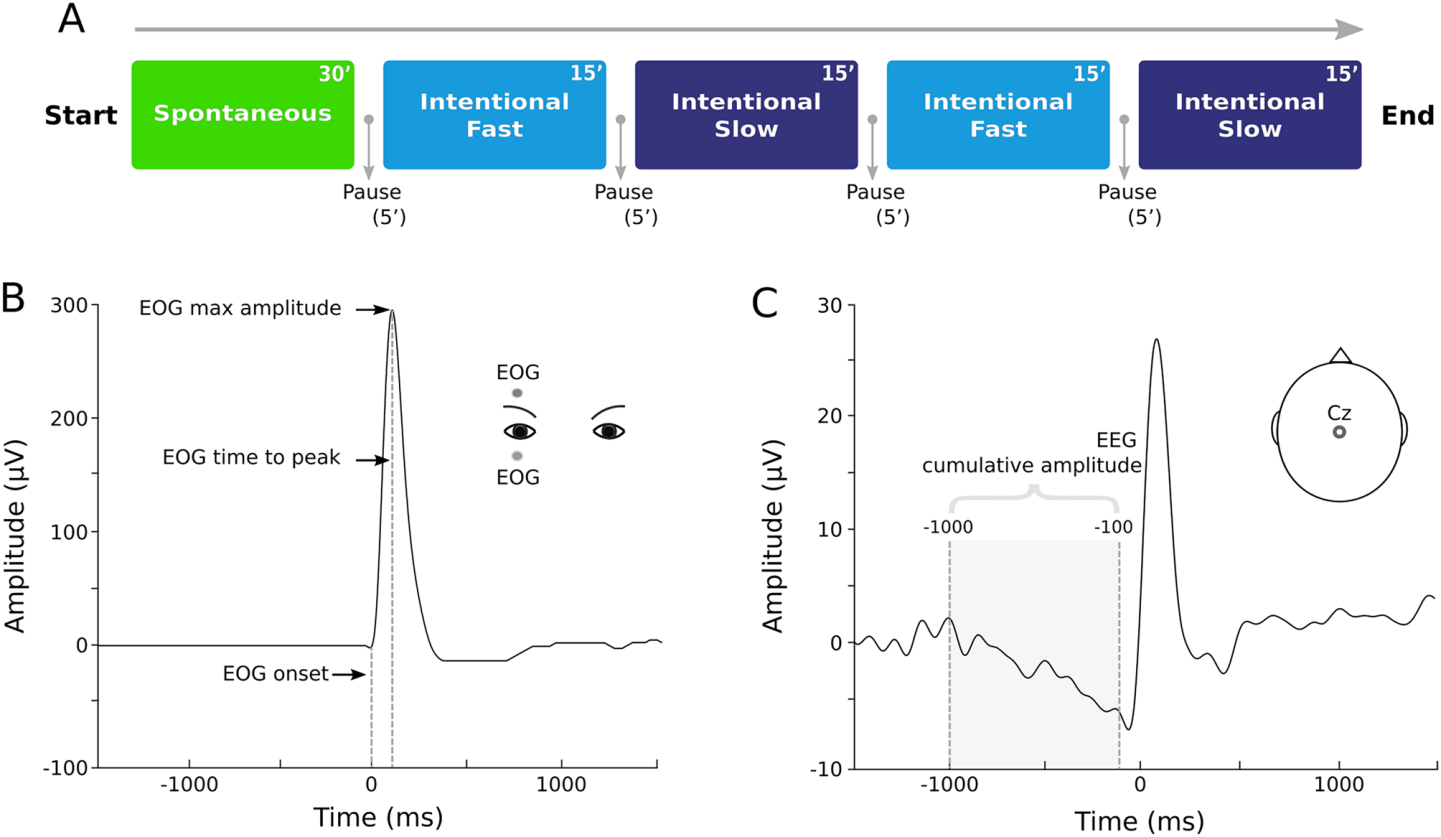
Outline of the experimental and analysis procedures. (**A**) Timeline of the different conditions of the experiment. (**B**) Representative time course of the electrical activity generated by a single blink, recorded by two EOG electrodes in bipolar montage positioned above and below the eyelid and aligned to the eye pupil in the primary central gaze position. The following measures were obtained from bipolar vertical EOG: EOG time to peak in ms (short vertical dashed line) and EOG max amplitude (long vertical dashed line). (**C**) Representative average EEG activity recorded from the Cz electrode around a blink (0 ms corresponds to the EOG onset). The cumulative amplitude from − 1 s to − 100 ms from EOG onset was computed as a functional EEG measure and was referred to as RP (Readiness Potential).

To address these issues we recorded the EOG and EEG activity from 17 healthy participants when blinking under three different conditions (Fig. 1).

In the first condition, participants did not receive any instruction, except for maintaining the gaze in a primary central position and staying relaxed avoiding muscle contractions (*Spontaneous, S*). In the second condition, they were asked to perform fast eye blinks at a self-paced rate (*Intentional Fast*, IF). In contrast, in the third condition, they were instructed to blink at a self-paced rate in a slow way as natural as possible, without focusing on the force to exert in the execution of eyelid movements (*Intentional Slow*, IS).

We considered two kinematic parameters i.e., the EOG amplitude and EOG time to peak^10^, as well as brain signals i.e., the RP computed as the EEG cumulative amplitude in a time window between − 1000 ms and − 100 ms before the EOG onset^15,18^.

The grand-average EEG waveform preceding intentional and spontaneous eye blinks showed that the RPs were widely distributed over the scalp (see, Supplementary Materials, Figure S1), with their amplitude being maximal in the midline electrode (Cz), in line with previous data^18^(but see^17^)**.**

**Figure 2:**
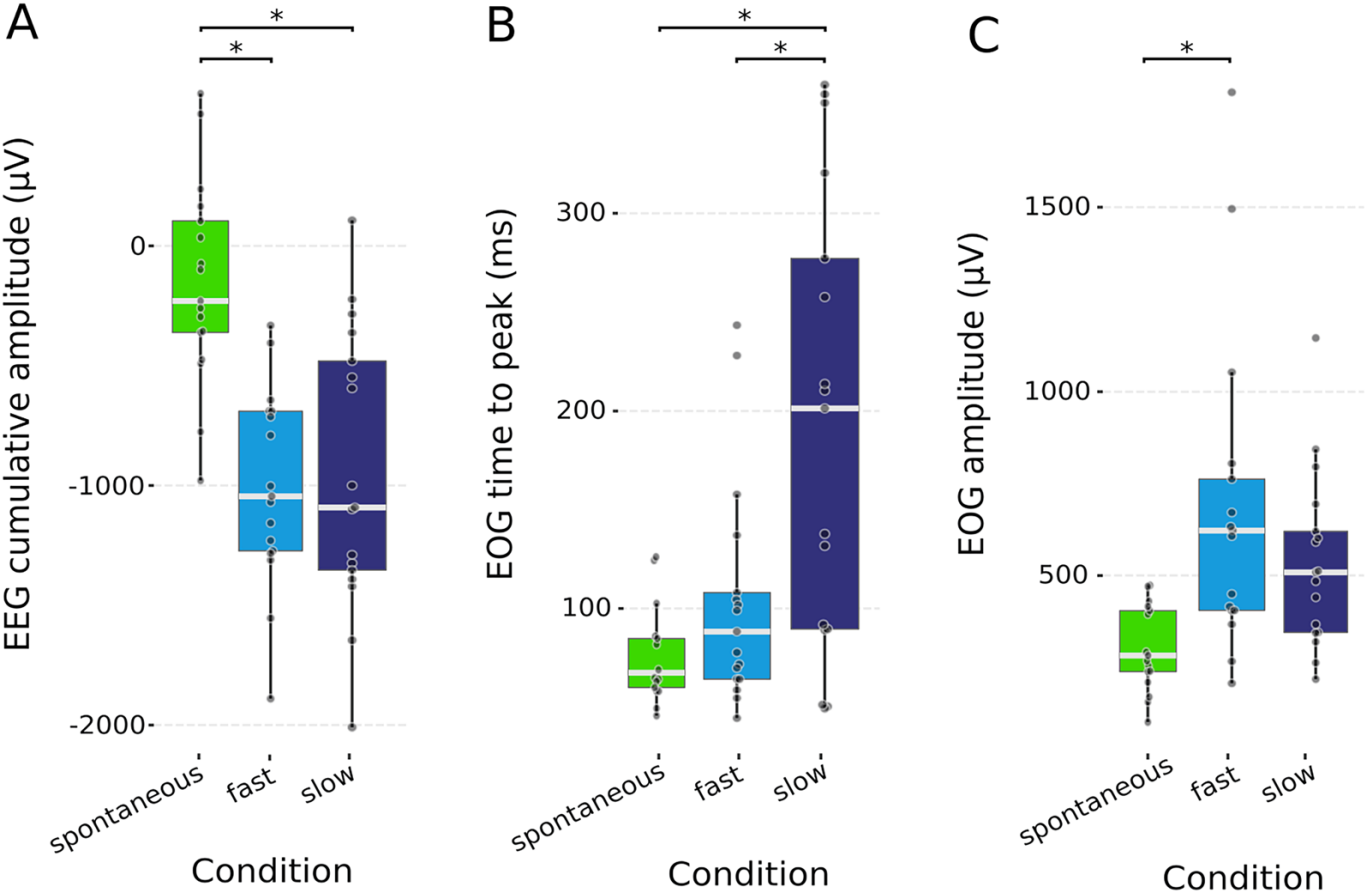
Comparison of EEG cumulative amplitude, EOG time to peak, and EOG amplitude across conditions. (**a**) EEG cumulative amplitude averaged across trials for all subjects, in spontaneous, fast, and slow intentional conditions (**b**) EOG time to peak averaged across trials for all subjects, in spontaneous, fast, and slow conditions. (**c**) EOG amplitude averaged across trials for all subjects in spontaneous, fast, and slow conditions.

To assess how the different markers were able to distinguish between intentional and spontaneous blinking, an analysis of variance was performed separately for the three dependent variables (EOG time to peak, EOG amplitude, and EEG cumulative amplitude) across the three conditions (S, IF, and IS). Results showed that EOG time to peak (F (2, 32) = 14.5, *p* = 0.00003, η2 = 0.32), EOG amplitude (F (2, 32) = 14.5, *p* = 0.00003, η2 = 0.23), and EEG cumulative amplitude (F (2, 32) = 26.43 *p* = 0.0000002, η2 = 0.41) were significantly different across conditions. However, post-hoc pairwise comparisons indicated that the EEG cumulative amplitude, but not the EOG time to peak and the EOG amplitude, systematically distinguished intentional from spontaneous blinking. Indeed, the EEG cumulative amplitude was larger in IF than in S (IF: mean =  − 1012 µV, sd = 403; S: mean =  − 158 µV, sd = 429; *p* = 0.000009); and was also larger in IS than in S (IS: mean =  − 942 µV, sd = 571, *p* = 0.00004). Interestingly, it did not significantly differ between the two intentional conditions IS and IF (*p* = 1). On the contrary, the EOG time to peak was larger in IS than in S (S: mean = 75.2 µV, sd = 23.5; IS: mean = 191 µV, sd = 112; *p* = 0.0001) and also larger in IS than in IF (IF: mean = 102 µV, sd = 57.1; *p* = 0.002), but it did not significantly differ between S and IF (*p* = 0.8). Finally, the EOG amplitude was significantly larger in IF than in S (S mean = 305 µV, sd = 114; IF mean = 684 µV, sd = 424, *p* = 0.001) while it did not significantly differ between S and IS (IS mean = 540 µV, sd = 234, *p* = 0.06) and between IS and IF (*p* = 0.4) (Fig. 2).

### The EEG marker significantly correlated with intentionality, but not with kinematics

To better understand the EEG contribution to the distinction between intentional and nonintentional actions, however, we adopted a more fine-grained approach. We analyzed the EEG amplitude when the eye blinks were *kinematically similar* but *different* in their *intentional nature* and when they were *kinematically different* but *similar* in their *intentional nature*. We, therefore, selected a number of trials of spontaneous and intentional blinking with a strong overlap in the EOG parameters and the same number of trials of intentional blinking with EOG parameters being radically divergent.

**Figure 3:**
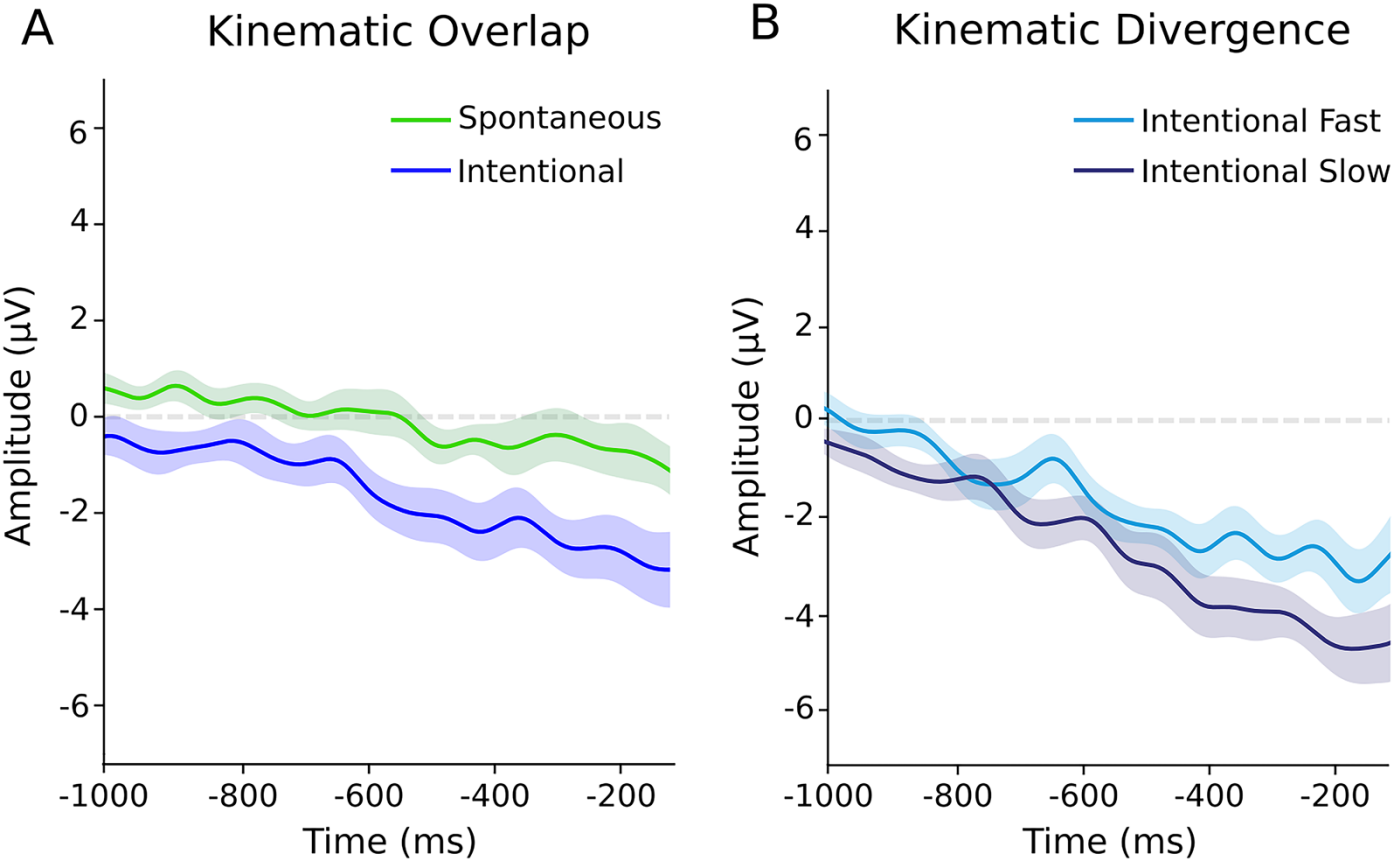
EEG cumulative amplitude of S and I (merging intentionally fast and slow) and of IF and IS in case of kinematic overlap and kinematic divergence. (**a**) Grand average of kinematically overlapped trials in S and I. (**b**) Grand average of kinematically divergent trials in the two intentional conditions: IF and IS.

The ANOVAs involving kinematically divergent and overlapping trials (Fig. 3; see "Methods" section) showed that the EEG amplitude significantly varied when the eye blinks differed in their being intentional (F (1,16) = 5.54, *p* = 0.03, η2 = 0.10), but not when they merely differed in kinematics (F (1,16) = 2.09, *p* = 0.16, η2 = 0.06). Indeed, the kinematically similar but intentionally different eye blinks were distinguishable in terms of EEG cumulative amplitude (Spontaneous: mean =  − 52.86 µV, sd = 538.05, Intentional: mean =  − 636.20 µV, sd = 805.24), while this was not the case for the intentionally similar but kinematically different eye blinks (Intentional Slow: mean =  − 760.50 µV, sd = 611.30, Intentional fast: mean =  − 1192.42 µV, sd = 720.55). (See Supplementary Materials, Figure S2 for their respective control analyses).

### EOG markers were not predictors of the EEG marker

As a further step, to rule out the possibility of dependence between the EEG amplitude and the EOG parameters we computed two standard linear regressions with EEG amplitude as the outcome and with each EOG parameter as the predictor (Supplementary Materials, Table 1). Results showed a nonsignificant relationship both for the EOG time to peak and the EEG cumulative amplitude (β = 0.25, SE = 0.38, F(1,18) = 0.43,*p* = 0.5, R^2^ = 0.02) and for the EOG amplitude and the EEG cumulative amplitude (β = 0.26, SE = 0.19, F(1,18) = 1.74, *p* = 0.2, R^2^ = 0.08).

### The EEG marker is a key predictor of intentional blinks

Next, we assessed how well models with different combinations of predictors (i.e. EOG time to peak, EOG amplitude, and EEG cumulative amplitude) were able to distinguish the two types of intentional blinks from the spontaneous condition. We employed a series of multinomial logistic regression analyses (see "Methods" section) which showed that the best model corresponded to the full model, which included all three markers (mean accuracy = 70.00%; mean mcAUC = 0.88, see Supplementary Materials, Table 2). The EEG cumulative amplitude was the only statistically significant predictor that could discriminate both between spontaneous and intentional slow blinks and between spontaneous and intentional fast blinks (*p* < 0.001 and *p* < 0.01, respectively). We then performed a logistic regression analysis grouping together the data from fast and slow intentional eye blinks into a unique intentional category and assessed how well models with different combinations of predictors were able to distinguish between the intentional and spontaneous conditions. The best model again corresponded to the full model (mean accuracy = 87.91%; mean AUC = 0.88). It is worth noting that the EEG cumulative amplitude was the only significant predictor (*p* < 0.01; see Supplementary Materials, Table 3), boosting the discrimination performance of the model from ~ 70% to above ~ 85% (see Supplementary Materials, Table 3).

### The full model applied to sessions of intentional and spontaneous blinks performed by three patients with brain injuries

As proof of principle, we then applied the latter model to data of three paradigmatic brain-injured patients with rare neurological syndromes characterized by motor and communicative impairments. Indeed, the first patient was affected by locked-in syndrome (LIS)^7–9^ resulting from an ischemic ponto-mesencephalic lesion, whereas the second patient suffered from akinetic mutism^19–21^ syndrome as a consequence of hemorrhagic bilateral damage of frontal lobes due to an anterior cerebral artery aneurysm rupture. Finally, the third patient was diagnosed as “minimally conscious state *plus*” (MCS +)^9^, which is a disorder of consciousness with a partially preserved albeit fluctuating command-following and an inability to functionally communicate (see Supplementary Materials, Table 4).

The model was able to correctly identify 12 sessions out of 14 as either intentional or spontaneous (acquired during a single day for Patient 1 and three consecutive days for Patient 2 and Patient 3). Two spontaneous sessions were incorrectly identified as intentional (see Supplementary Materials, Table 5).

## Discussion

The main aim of the present study was to investigate the distinction between intentional and nonintentional actions by taking advantage of brain-based and kinematic markers. In doing this we contrasted intentional and spontaneous eye blinking. Spontaneous eye blinks can be generated endogenously, without the need for external triggers. This sets them apart from reflexes and other externally driven movements, which are typically considered nonintentional. Spontaneous blinks are similar in this respect to intentional eye blinks, and are thus a more ideal control condition compared to other no-intentional movements commonly studied.

The first main finding of this study was that the EEG but not the EOG markers systematically distinguished intentional from spontaneous blinking. Indeed, the same eye blinks were preceded by a different brain activity depending on whether they were intentional or nonintentional. Unlike spontaneous ones, intentional blinks were preceded by a slow negative EEG drift, which has been usually construed in terms of RP^18,22^, regardless of their fast or slow execution.

This finding is in line with some previous pieces of evidence. In a pioneering study, Montagna and Zucconi (1984) recorded EEG activity before spontaneous and intentional eye blinks. Their results showed that intentional, but not nonintentional, eye blinks were preceded by a cortical RP^16^. Kaneko et al*.* (2004) and, more recently, Mota and Lins (2017) replicated these results, by showing intentional eye blinks to be prefaced by a negative potential^12,17^.

However, an important limitation of these studies is that they did not control the role of kinematics. Indeed, manipulating performance profiles is critical for elucidating the neuronal substrates of intentional and nonintentional actions as intentional eye blinks may purposefully take very different kinematic profiles. For instance, you can intentionally blink very quickly or very slowly. The difference in velocity alters the performance, but not the intentional nature of the action.

To overcome this limitation, we designed our study to compare similarities and differences in kinematics and intentionality; to do so, we analyzed the EEG activity both before spontaneous and intentional eye blinks with a strong overlap in the kinematic parameters and before intentional eye blinks with the kinematics parameters being radically divergent. We found the EEG amplitude to significantly vary when eye blinks differed in their being intentional, but not when they merely differed in their kinematics. Crucially, the EEG amplitude distinguished intentional from nonintentional eye blinks even when they were kinematically similar; on the contrary, it did not differentiate the eye blinks when they all were intentional but kinematically different.

Taken together, these findings indicate that EEG signals preceding the onset of the movement may critically contribute to distinguishing intentional from nonintentional blinking, regardless of their kinematic profiles. Attributing a distinctive role to the RP in distinguishing intentional from nonintentional action does not require any commitment to the existence of distinct states or processes causally related to the occurrence of action, such as intention, will, or associated philosophical paraphernalia^23^.

Stochastic decision models have emphasized this point, proposing that the readiness potential (RP) reflects ongoing stochastic fluctuations in neural activity that result in movement initiation only after crossing a specific motor threshold^24,25^. These fluctuations manifest as a slow buildup in the movement-locked average but do not necessarily indicate any purposeful or intentional preparation for movement^26^.

However, our findings introduce an additional layer of complexity to the stochastic decision model framework. The failure to record an RP systematically preceding spontaneous blinking indeed represents an opportunity to deepen our understanding of the relationship between ongoing stochastic fluctuations at the cortical level and action initiation. Indeed, even if spontaneous blinks are thought to be subcortically generated^12,27^, their rate of occurrence has been shown to vary dramatically depending on cognitive and attentional demands^28–33^. Since the processes are cortically mediated^34–37^, one would expect the timing of spontaneous blinking to be significantly biased by stochastic fluctuations in cortical excitability. The absence of a significant slow drift in the average EEG activity before spontaneous blinking is thus an interesting result that should be considered in the general debate about the nature of the RP.

Theoretical implications aside, our findings also have practical implications as they suggest a way to improve the discrimination between intentional and nonintentional actions irrespective of overt communication, by integrating EEG and kinematic markers. We first tested this hypothesis in healthy subjects by running a series of multinomial logistic regression analyses. As expected, the best model corresponded to the full model, which included both the EEG and EOG markers. Interestingly, the EEG marker was the only statistically significant predictor that could discriminate between spontaneous and intentional blinks. We then performed a logistic regression analysis grouping together fast and slow intentional eye blinks into a unique intentional category. Again, the best model corresponded to the full model, and the EEG marker was the only significant predictor. These results indicate that combining EEG and EOG provides the best discriminatory performance between intentional and unintentional blinks, and suggest that future attempts to develop clinical tools based on blinking should consider integrating them.

Finally, as proof of principle, we applied this model to data from three paradigmatic brain-injured patients with rare neurological syndromes. Patients were characterized by a different degree of impairment of motor action generation, ranging from the absence of intentional limb movements due to strategic pyramidal tracts lesion (locked-in syndrome) or to a failure to initiate motor responses despite a preserved intrinsic capacity to move (akinetic mutism syndrome) up to fluctuating motor responsiveness in the context of a disorder of consciousness (minimally conscious state plus).

The model was able to correctly identify 12 sessions out of 14 as either intentional or spontaneous. Two spontaneous sessions were incorrectly identified as intentional. The first spontaneous session that was misidentified as intentional, was recorded in the LIS patient in the very acute setting (ICU). In this phase, the patient could blink intentionally, since it was the only act he could perform. It is thus likely that some blinks were intentional even during the spontaneous session. The second mismatch occurred in the minimally conscious state *plus* (MCS +) patient during a spontaneous session that followed an intentional session (the day before, see "Methods" section—Patients), and hence in a condition where the patient was no longer naive to the experimental procedure and also can be ascribed to her/his motor perseveration symptoms.

In patients with severe brain injuries blinking is often the only motor action that can be reliably detected. Finding a brain-based way of knowing whether a patient’s blinks are nonintentional events or intentionally driven is of crucial importance. Usually, in patients with a disorder of consciousness, intentionality is assessed based solely on behavioral examinations (see, Supplementary Materials, Table 5), asking patients to perform simple actions such as visual pursuit or more complex actions such as functional use of an object. In the past few years, several EEG-related measures were used to try to assess residual consciousness after severe brain injury, such as event-related potentials (ERPs) in different modalities, quantitative EEG (qEEG), transcranial magnetic stimulation (TMS) combined with EEG (TMS/EEG), and active paradigms with either fMRI and EEG^38^. In the context of a multidimensional assessment of residual cognitive capacity upon recovery of consciousness^39–41^, a simple set-up of two EOG derivations and one EEG channel (Cz) may provide a dependable and fast readout to probe intentionality at the bedside.

### Limitations of the study

The very small number of patients and sessions did not allow us to draw any robust inference about the effective power of the model as well as the actual contribution of each marker (kinematic, EEG) to distinguishing between intentional and spontaneous blinking. However, these results represent a promising proof of principle corroborating the notion that adding brain-based measures to kinematics provides useful information for discriminating intentional from nonintentional actions in patients suffering from motor and communicative impairments.

## Methods

### Healthy participants

Seventeen (17) *naïve* healthy subjects (mean age: 28 years, sd: 4.4 years, 8 females**)** were tested at the Department of Biomedical and Clinical Sciences “L. Sacco” of the University of Milan. All participants gave their written informed consent to take part in the experiment, according to the Declaration of Helsinki. All participants had normal or corrected to normal visual acuity and did not wear glasses or contact lenses during the experiment. The experimental procedure was approved by the Ethics Committee of the Ospedale “L. Sacco” (Milano Area 1), Milan, Italy.

### Patients

Three (3) brain-injured patients with different clinical conditions were tested (Supplementary Materials, Table 4). Patient 1 was recorded at “San Gerardo” Hospital in the Intensive Care Unit (ICU), Monza, Italy. Patient 2 and Patient 3 were recorded in a rehabilitation facility of Fondazione Don Carlo Gnocchi ONLUS, Milan, Italy. The informed consent was acquired from legal surrogates. The following ethical Committee approved all experimental procedures: *Comitato etico della provincia Monza e Brianza*, Milan, Italy, and *Fondazione Don Gnocchi—Istituto di Ricovero e Cura a Carattere Scientifico Fondazione Don Gnocchi Onlus*, Milan, Italy.

## Method details

### Experimental design

#### Healthy participants

Participants were seated in a comfortable chair and were requested to look straight at a dark screen set at a 1.5 m distance to limit eye movements (i.e. saccades). They were instructed to maintain the gaze in a central position and to avoid body and head movements as well as facial muscle contractions.

The experiment consisted of three conditions: (i) *Spontaneous* (S); (ii) *Intentional Fast* (IF), and iii) *Intentional Slow* (IS). The three conditions were recorded on the same day.

The S condition was always recorded first to maintain the participants unaware of the objective of the study and to prevent them from focusing their attention on the eyelid movements related to the execution of spontaneous eye blinks. During S, the underlying assumption was that participants performed only non-intentional eye blinks since they were *naïve* concerning the aim of the study. In IF, participants were asked to intentionally perform “fast” eye-blinks, i.e., paying attention to the effort made in execution. In IS, participants were instructed to intentionally blink in a “slow” way, as naturally as possible, without focusing on the exerted force. Both for IF and IS, participants were instructed to perform intentional eye-blinks at a self-paced rate (i.e. without cueing signals); however, a particular emphasis was put on the requests of keeping a moderate separation (i.e. at least one second) between two consecutive movements and avoiding counting.

IF and IS were alternated to prevent habituation. At the beginning of the first IF and IS session, participants were shortly trained on how to properly perform the aforementioned movements to minimize the task-related cognitive efforts that might have an impact on the EEG cumulative amplitude. We also allowed for short breaks in-between sessions to prevent tiredness.

In the first 6 subjects, we recorded up to 8 sessions of 15 min per condition, to be able to assess the number of trials required for obtaining reliable RPs without inducing fatigue. From this group, we estimated that the RP stabilized after averaging ~ 30 trials (see Supplementary Materials, Figure S3). For this reason, in the remaining 11 subjects we recorded from 1 to 3 sessions of 15 min of each condition (S, IF, IS) in order to record at least 30 trials for each one of them and to minimize tiredness and fatigue due to the global duration of the recordings.

#### Patients

Patients were seated in a wheelchair in a dedicated room, in order to minimize discomfort due to environmental noise.

The experiment consisted of two different conditions: (i) Spontaneous (S); (ii) Intentional (I) acquired all on the same day. In the case of patients, we limited the exploration to the contrast between spontaneous and intentional blinking, without explicitly asking them to perform intentional eye blinks quickly or slowly, because this was unfeasible due to attentional, aphasic, or motivational deficits possibly related to their specific neurological syndrome and fatigue.

In the S condition blinks were recorded in the same way as in healthy participants. In the I condition a trained neuropsychologist repeatedly asked patients to perform intentional eye blinks. A marker was manually inserted on the continuous EEG recording whenever the neuropsychologist asked the patient to perform an eye blink. In this way, blinks performed immediately after the request were identified. As for the healthy participants, we recorded from 1 to 3 sessions of 15 min for each condition (S and I). We repeated session acquisitions for Patient 2 and Patient 3 during three consecutive days to better capture patients’ reactivity. For Patient 1, due to the peculiarity of the experimental setup (ICU), it was not possible to repeat the acquisition in the following days after the first acquisition.

#### EEG acquisition (healthy participants and patients)

EEG data was recorded using a 64-channel EEG amplifier (BrainAmp DC, Brain Products, Germany). EEG was recorded from 62 surface electrodes placed in a cap according to the International 10/20 System. Impedances were kept below 5 kΩ and the signal was acquired at a sampling rate of 5000 Hz. All electrodes were referenced to the linked earlobes and a ground electrode was placed on the forehead. A pair of additional electrodes were placed above and below the right eye in line with the pupil when the gaze was in the central position. The distance between the two electrodes was kept constant between subjects and was approximately 85 mm^10^, considering individual anatomical differences. A vertical electrooculogram (EOG) was obtained by computing the difference (above–below) between these two electrodes.

#### Eye blink detection (healthy participants and patients)

Data analysis was performed using Matlab R2015b (Mathworks Inc.) and custom-made scripts based on EEGLab. Eye blinks were identified offline on band-pass filtered bipolar EOG derivations (0.01 Hz: 1st order finite impulse response; 10 Hz: 3rd order Butterworth) to attenuate slow drifts. Eye-blink onset was set at the time of occurrence of the positive peaks of the first derivative of the EOG. EOG epochs, between − 2 s to 2 s and centered on the eye-blink onset, were visually inspected, and epochs with either bad signal quality or with more than one eye-blink were discarded.

#### EOG analysis (healthy participants and patients)

The kinematic features of eye blinks were characterized by computing their amplitude and time to peak on the EOG signal^10^. The continuous EOG signal was band-pass filtered between 0.1–8 Hz and subsequently segmented into − 2 s to 2 s time windows around the eye-blink’s onset. EOG epochs were baseline corrected in the time interval from − 1 s to − 0.1 s*.* Blink amplitude was calculated as the peak value of the EOG starting from the blink’s onset time. The onset time was detected as the first value exceeding three standard deviations from the baseline. Blink time to peak was calculated as the time, in ms, from the onset to the peak.

#### EEG analysis (healthy participants and patients)

The continuous EEG signal was filtered (band-pass 0.1–8 Hz), segmented between − 2 s to 2 s around the blink’s onset (obtained from the EOG), and down-sampled to 500 Hz. Epochs belonging to the same condition (S, IF, IS) were grouped across sessions. The time interval from − 1.5 s to − 1 s was used for baseline correction.

Epochs were visually inspected to reject segments contaminated by muscular artifacts or line drifts due to bad impedances. In the healthy participants, the mean number of good epochs we obtained after trial rejection was 128 for S (sd: 67, min: 46, max: 254, total number: 2177), 152 for IF (sd: 74, min: 45, max: 285, total number: 2589), and 151 for IS (sd: 75, min: 65, max: 321, total number: 2574). In patients, the mean number of good epochs, after trial rejection, was 72 for S (sd: 70, min: 26, max: 219) and 51 for I (sd: 36, min: 33, max: 126).

EEG analyses were focused on the signal preceding eye-blink onset where the Readiness Potential is known to occur. Data for statistical testing was obtained from *Cz* since previous evidence has shown that the Readiness Potential is maximal at this site^18^.

To quantify the *Readiness Potential*, we chose the *cumulative amplitude*^42^, calculated as the total sum of values in *Cz* in a window between − 1 s and − 100 ms before the blink’s onset. We did not include the last 100 ms to avoid blink-related drifts.

## Statistical analysis

### Healthy participants

All statistical analyses were performed using the R software^43^. Results of specific tests with summary statistics and information on the test used are reported in the Results section. In all the analyses performed, values were averaged across trials for each subject and condition using the 20% trimmed mean to avoid possible influences of outliers^44^. Multiple comparison corrections were performed using the Holm-Bonferroni method. For the sample size justification please see Supplementary Figure S4.

### Differences in EEG cumulative amplitude, EOG time to peak, and EOG amplitude in SC, IFC, and ISC

Our first aim was to understand the differences between S, IF, and IS, in the EEG cumulative amplitude, the EOG time to peak, and the EOG amplitude. To this aim, we performed an analysis of variance for each of the three dependent variables (EEG cumulative amplitude, EOG time to peak, EOG amplitude) across conditions.

### Kinematic pairing

Our second aim was to investigate the two following interconnected issues. First, we evaluated whether the EEG cumulative amplitude changes between intentional and spontaneous conditions when the kinematics parameters of the two conditions overlap, that is, when they have similar kinematic features. Second, we addressed the issue of whether the EEG cumulative amplitude varies within intentional conditions (IF and IS) when the kinematics parameters of the two conditions diverge.

Regarding the first aim, for each subject, we averaged trials (see below) from the spontaneous condition on one hand and from the intentional conditions on the other, with an overlap in their kinematic parameters (EOG amplitude and EOG time-to-peak), and compared them using a paired t-test. Regarding the second aim, for each subject, we averaged trials from the fast condition and the slow condition, with divergent kinematic parameters, and compared them using a paired t-test. Kinematic overlap and kinematic divergence were defined by the Mahalanobis Distance^45^, which is a multivariate metric based on the distance between a point and a distribution. Specifically, for the overlap analysis we extracted the 30 trials per subject of the intentional conditions that were closer to the centroid of the distribution of the S condition and the 30 trials of the S condition that were also closer to the centroid of the S distribution (i.e. most representative of the spontaneous condition), to verify the presence of an RP in the intentional trials whose kinematics were similar to those of spontaneous trials. Conversely, for the divergence analysis, we extracted the 30 trials per subject of each intentional condition that were more distant from the other intentional condition. An additional criterion was used to ensure that trials were either overlapping or divergent according to the case. Intentional trials overlapping with the spontaneous condition trials were used if they were within the 95th percentile of the distribution of distances of the spontaneous condition, and divergent intentional trials were used if they were beyond the 70th percentile of the other intentional condition. See the kinematic overlap and divergence section in the Supplementary Materials (Figure S2) for a summary of the control tests performed on the retained samples and graphical representations.

### EEG vs kinematics

Regarding the third aim, to control that the EEG cumulative amplitude did not depend on the magnitude of the kinematic parameters we considered the EEG as the outcome variable and computed two standard linear regressions, first with the EOG time to peak and second with the EOG amplitude as a predictor. In both cases, predictors were grouped in quantiles (20 groups) and we tested if it was possible to predict the EEG from the kinematic variables (Supplementary Materials, Table 1).

### Classification of spontaneous and intentional sessions

Our fourth aim was to understand which combination of variables (EEG cumulative amplitude, EOG time to peak, and EOG amplitude) better predicted if a specific session belonged to a spontaneous or intentional condition. For each subject and condition, we computed the 20% trimmed mean of each variable to reduce possible influences of outliers and conducted two different sets of analyses.

First, to evaluate how well each variable could differentiate between the spontaneous condition and each type of intentional blink we employed a multinomial logistic regression. The dependent variable corresponded to blink type (spontaneous, fast, and slow; with spontaneous as the reference level) see Supplementary Material (Table 2). We postulated four competing models: the full model, which included all three predictors (EEG cumulative amplitude, EOG amplitude, and EOG time-to-peak), and three other models in which one of the predictors was dropped and the other two retained (EEG cumulative amplitude and EEG time-to-peak; EEG cumulative amplitude and EOG amplitude; EOG amplitude and EOG time-to-peak). Model selection was performed using the Akaike Information Criterion (AIC)^46^. We assessed the statistical significance of the predictors on the best model using the Wald z-test, and Holm-Bonferroni multiple comparisons correction. Model performance was assessed by computing the discrimination accuracy (percentage of correct classification) with 20-fold cross-validation (75% train-test splitting), and the multiclass Area Under the Receiver Operating Characteristic Curve (mcAUROC)^47^.

Second, we grouped the data from the fast and slow conditions into a unique intentional category; we used spontaneous conditions as a reference level and performed a logistic regression analysis (Supplementary Material, Table 3). We postulated the same four competing models as in the previous analysis. Model selection was again performed using AIC and model performance was assessed by computing the discrimination accuracy (percentage of correct classification) with 20-fold cross-validation (75% train-test splitting) with up-sampling (on each cross-validation iteration, with replacement), and the Area Under the Receiver Operating Characteristic Curve (AUROC). Statistical significance of the predictors on the best model was tested using the Wald z-test, and Holm-Bonferroni multiple comparisons correction.

### Patients

The full model (EEG cumulative amplitude, EOG amplitude, and EOG time-to-peak) was employed in patients’ 14 sessions to obtain predictions of the conditions under which the eye blinks were performed. The model’s efficacy was tested on S and I conditions.

## Data and code availability

The datasets used and/or analyzed during the current study are available from the corresponding author upon reasonable request.

## Supplementary Information

Supplementary Information 1.

Supplementary Information 2.

Supplementary Information 3.

Supplementary Information 4.

Supplementary Information 5.
