# Supplementary figures and images for: Distinguishing intentional from nonintentional actions through eeg and kinematic markers
Source: Sci Rep. 2023 May 25;13:8496. doi: 10.1038/s41598-023-34604-y (PMC10213007; doi:10.1038/s41598-023-34604-y)

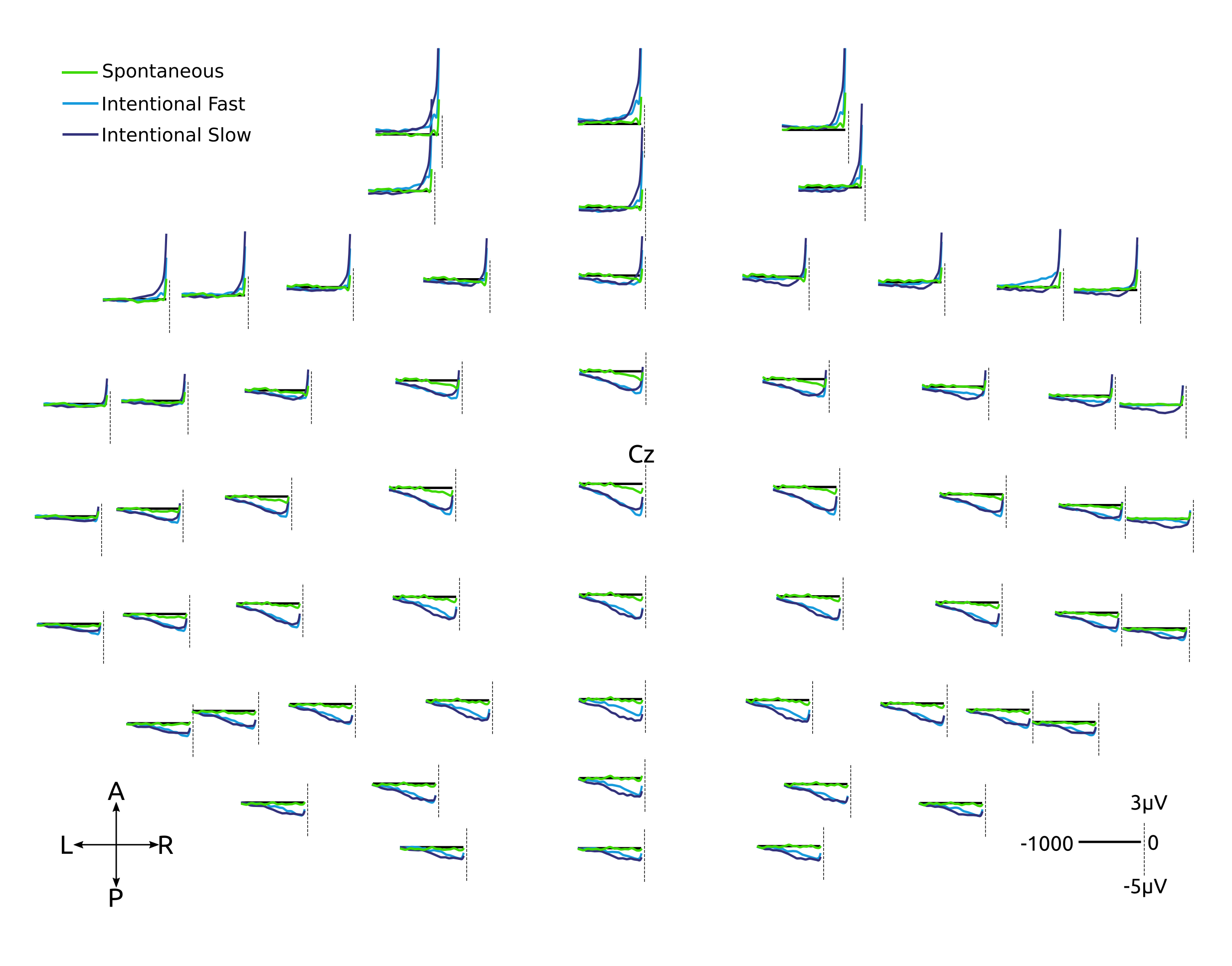

Supplement: Supplementary file 1 — Supplementary Information 1. [file 41598_2023_34604_MOESM1_ESM.tiff]

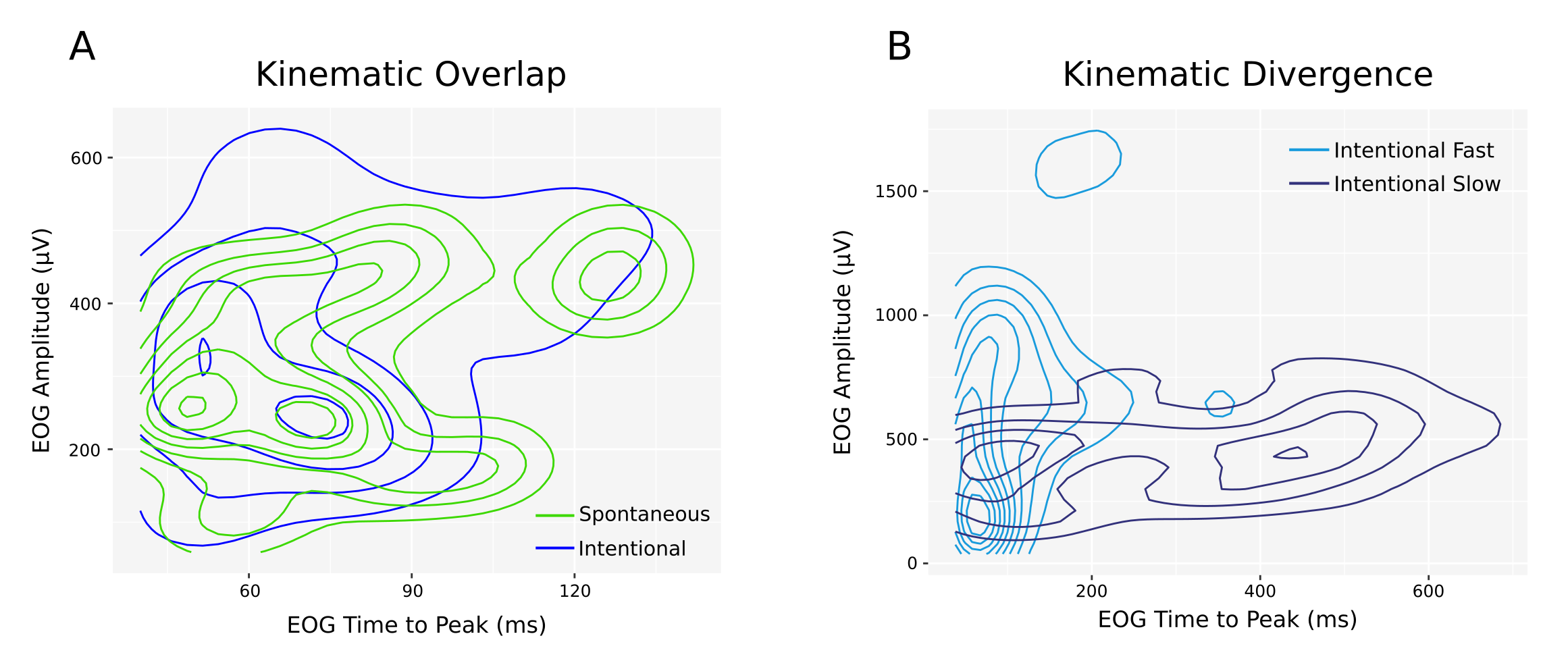

Supplement: Supplementary file 2 — Supplementary Information 2. [file 41598_2023_34604_MOESM2_ESM.tiff]

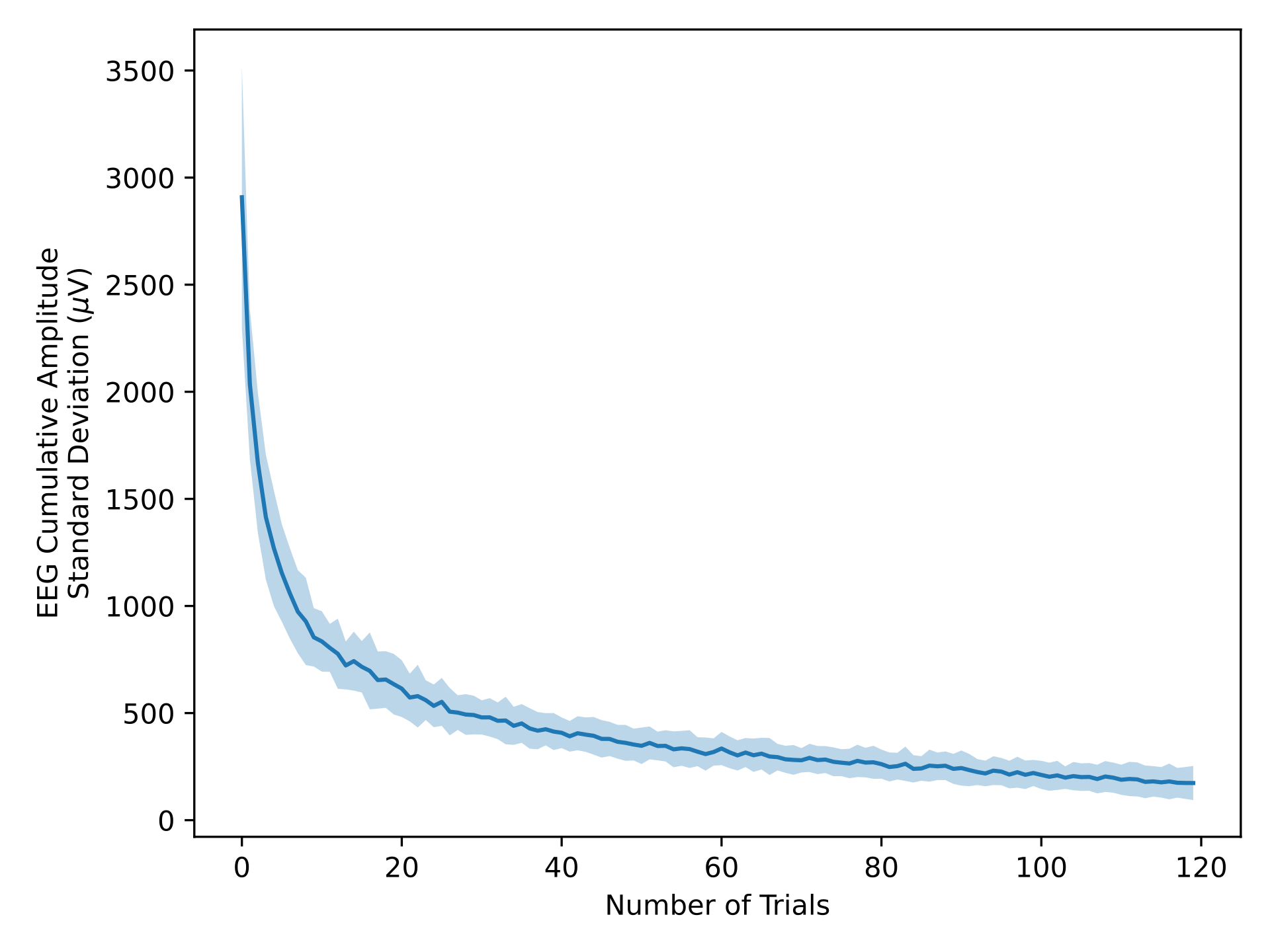

Supplement: Supplementary file 3 — Supplementary Information 3. [file 41598_2023_34604_MOESM3_ESM.tiff]

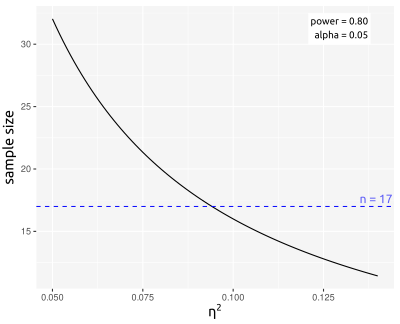

Supplement: Supplementary file 4 — Supplementary Information 4. [file 41598_2023_34604_MOESM4_ESM.tiff]
