# Supplementary material for: Distinguishing intentional from nonintentional actions through eeg and kinematic markers
Source: Sci Rep. 2023 May 25;13:8496. doi: 10.1038/s41598-023-34604-y (PMC10213007; doi:10.1038/s41598-023-34604-y)
Supplement: Supplementary file 5 — Supplementary Information 5. [file 41598_2023_34604_MOESM5_ESM.docx]

**SUPPLEMENTARY MATERIALS**

**Supplementary Figure S1. Topographical map of EEG cumulative amplitude.**

**Figure S1.** EEG activity is shown in all channels from -1000 to 0 ms (all trials averaged) corresponding to blink onset in the three conditions (Spontaneous: green; Intentional Fast: light blue; Intentional Slow: dark blue). The RP (i.e., the cumulative EEG amplitude between -1s and -100 ms) is maximally represented around the Cz site. A/P= anterior/posterior, L/R= left/right.

**Supplementary Figure 2. Control tests for kinematic overlap and kinematic divergence.**

Two independent-sample Welch t-tests were conducted to compare the EOG time to peak and amplitude between intentional and spontaneous conditions matched by kinematic overlap. There was no significant difference in time to peak between the intentional (M = 85.35, SD = 40.6) and spontaneous (M = 75.04, SD = 24.9) conditions (t(26.53) = 0.8, p = 0.38). Also regarding amplitude, there was no significant difference between the intentional (M = 376.73, SD = 147) and spontaneous (M = 305.37, SD = 115) conditions(t(30.16) = 1.57, p = 0.12). Another two independent-sample Welch t-tests were conducted to compare time to peak and amplitude between fast and slow sessions matched by kinematic divergence. There was a significant difference in time to peak between fast (M = 124.62, SD = 95.2) and slow (M = 308.44 SD = 182) sessions (t(24.11) = -3.68, p = 0.0005). Amplitude was also significantly higher in fast (M = 787.65, SD = 625) than in slow sessions (M = 482, 86, SD = 169; t(18.32) = 1.94, p = 0.03).

**Figure S2**. Kinematic Pairing. Two-dimensional histograms are represented as contour plots of the kinematic characteristics of the trials used for the kinematic pairing analyses. A) Distributions of the trials used for the kinematic overlap analysis. B) Distributions of the trials used for the Kinematic Divergence analysis.

**Supplementary Figure 3. Visualization of EEG cumulative amplitude variability related to the number of trials for each participant.**

**Figure S3**. The standard deviation of the EEG cumulative amplitude as a function of trial numerosity used for determining the minimum number of trials to be used to obtain a reliable RP. The blue line and shaded area represent the mean and standard deviation of the EEG cumulative amplitude standard deviation across 50 permutations in which the number of trials represented on the x-axis were randomly sampled from each subject. The graph shows that after approximately 30 trials the estimated value stabilizes and therefore it was set as the minimum number of trials per subject to use in the analyses.

**Supplementary Figure 4. Sample size justification.**

**Figure S4**. Power analysis showing the sample size as a function of the effect size (η2; with power = 0.8 and alpha = 0.05) for repeated measures ANOVA. The dashed blue line represents the actual sample size of the study.

**Supplementary Table 1. Table of Standard Linear Regression: data grouped in quantiles (20 groups).**

**Residuals**

| **Min** | **1Q** | Median | **3Q** | Max |
| --- | --- | --- | --- | --- |
| -408.99 | -86.19 | -10.29 | 107.44 | 424.94 |

**Coefficients**

| **Term** | **Estimate (exp)** | **Std. Error** | **t-value** | **p-value** |
| --- | --- | --- | --- | --- |
| Intercept | -883.0456 | 75.8237 | -11.646 | 0.00000000008 |
| Time to peak | -0.2513 | 0.3819 | -0.658 | 0.519 |

| **Residual standard error** | **Df** | **Multiple R-squared** | Adjusted R-squared | F | p-value |
| --- | --- | --- | --- | --- | --- |
| 212.3 | 18 | 0.02348 | -0.03077 | 0.4329 | 0.5189 |

**Residuals**

| **Min** | **1Q** | Median | **3Q** | Max |
| --- | --- | --- | --- | --- |
| -425.37 | -143.37 | -50.77 | 165.56 | 611.68 |

**Coefficients**

| **Term** | **Estimate (exp)** | **Std. Error** | **t-value** | **p-value** |
| --- | --- | --- | --- | --- |
| Intercept | -779.4422 | 126.3622 | -6.168 | 0.000008 |
| Amplitude | -0.2601 | 0.1966 | -1.323 | 0.203 |

| **Residual standard error** | **df** | **Multiple R-squared** | Adjusted R-squared | F | p-value |
| --- | --- | --- | --- | --- | --- |
| 280.1 | 18 | 0.08858 | -0.03795 | 1.749 | 0.2025 |

**Supplementary Table 2. Multinomial logistic regression: spontaneous, intentional slow and intentional fast conditions.**

A. Model Performance

| **Model** | **df** | **AIC** | **Discrimination Accuracy (Mean)** | **Discrimination Accuracy**  **(SD)** | **Classification mcAUC**  **(Mean)** | **Classification mcAUC**  **(SD)** |
| --- | --- | --- | --- | --- | --- | --- |
| Full | 8 | 68.64 | 70 | 8.71 | 0.88 | 0.08 |
| EEG cumulative amplitude + EOG time-to-peak | 6 | 73.82 | 69.16 | 10.1 | 0.84 | 0.09 |
| EEG cumulative amplitude + EOG amplitude | 6 | 82.88 | 52.08 | 11.7 | 0.76 | 0.08 |
| EOG time-to-peak + EOG amplitude | 6 | 87.45 | 59.5 | 11.8 | 0.83 | 0.09 |

B. Coefficients (exp)

| **Condition** | **(Intercept)** | **EEG cumulative amplitude** | **EOG time-to-peak** | **EOG amplitude** |
| --- | --- | --- | --- | --- |
| Slow | 0.0000291 | 0.9942 | 1.0516 | 1.005911 |
| Fast | 0.0001591 | 0.9955 | 1.0307 | 1.009857 |

C. p-values (uncorrected)

| **Condition** | **EEG cumulative amplitude** | **EOG time-to-peak** | **EOG amplitude** |
| --- | --- | --- | --- |
| Slow | 0.0000229 | 0.01609 | 0.244 |
| Fast | 0.0002272 | 0.14299 | 0.049 |

D. p-values (corrected)

| **Condition** | **EEG cumulative amplitude** | **EOG time-to-peak** | **EOG amplitude** |
| --- | --- | --- | --- |
| Slow | 0.0001371 | 0.064 | 0.2859 |
| Fast | 0.0011 | 0.285 | 0.1498 |

E. Contingency Table

|  | Condition | Condition | Condition |
| --- | --- | --- | --- |
| Predicted | Spontaneous | Slow | Fast |
| Spontaneous | 16 | 1 | 2 |
| Slow | 0 | 11 | 2 |
| Fast | 1 | 5 | 13 |

**Supplementary Table 3. Logistic regression: spontaneous and intentional conditions.**

A. Model Performance

| **Model** | **df** | **AIC** | **Discrimination Accuracy (Mean)** | **Discrimination Accuracy (SD)** | **Classification AUC**  **(Mean)** | **Classification AUC**  **(SD)** |
| --- | --- | --- | --- | --- | --- | --- |
| Full | 4 | 27.89 | 87.91 | 9.54 | 0.88 | 0.12 |
| EEG cumulative amplitude + EOG time-to-peak | 3 | 28.89 | 86.25 | 9.47 | 0.87 | 0.11 |
| EEG cumulative amplitude + EOG amplitude | 3 | 31.40 | 76.24 | 7.77 | 0.84 | 0.12 |
| EOG time-to-peak + EOG amplitude | 3 | 47.67 | 66.66 | 8.96 | 0.73 | 0.12 |
| EEG cumulative amplitude |  |  | 70.41 | 8.74 | 0.74 | 0.12 |

B. Coefficients

| **Term** | **Estimate (exp)** | **Std. Error** | **z-value** | **p-value** |
| --- | --- | --- | --- | --- |
| Intercept | -8.632244 | 3.19 | -2.699 | 0.00695** |
| EEG cumulative amplitude | -0.004903 | 0.001 | -2.949 | 0.00319** |
| EOG time-to-peak | 0.038046 | 0.026 | 1.455 | 0.14571 |
| EOG amplitude | 0.008289 | 0.005 | 1.462 | 0.14374 |

C. Contingency Table

|  | Condition | Condition |
| --- | --- | --- |
| Predicted | spontaneous | intentional |
| spontaneous | 17 | 4 |
| Intentional | 0 | 30 |

**Supplementary Table 4. Patients’ Diagnosis and Coma Recovery Scale-Revised (CRS-r)^48^ scores.**

| Patient ID | Patient 1 | **Patient 2** | **Patient 3** |
| --- | --- | --- | --- |
| Diagnosis | **Locked-in syndrome** | **Akinetic Mutism syndrome** | **Minimally conscious state *plus*** |
| CRS-r total score | 20 | 18 | 15 |
| Auditory | 4 | 4 | 4 |
| Visual | 5 | 5 | 5 |
| Motor | 5 | 3 | 2 |
| Oromotor/verbal | 1 | 1 | 2 |
| Communication | 2 | 2 | 0 |
| Arousal | 3 | 3 | 2 |

**Patients**

***Patient 1*** was diagnosed as locked-in syndrome (LIS) that is defined by the presence of reproducible voluntary eye-opening and closing, preserved consciousness, aphonia, quadriplegia or quadriparesis and a primary mode of communication that uses vertical eye movement or blinking. This state just superficially resembled the vegetative state: the patient appeared extremely impaired at the level of motor behavior output, but he was able to communicate effectively through spared blinking only. In this case, blinking was used as a channel to communicate. ***Patient 2*** was characterized by a severe frontal syndrome known as *akinetic mutism* which refers to a particular clinical condition in which the patient, although awake and conscious, typically remains silent (mutism) and motionless (akinetic). This generalized lack of limb movements does not depend on specific damage along the pyramidal tract (as in the LIS) but represents the extreme along a spectrum of frontal symptoms of diminished motivation such as abulia (i.e., lack of will and motor initiative) and apathy (i.e. lack of emotional involvement). This specific neurologic disorder is typically caused by lesions involving the bilateral medial frontal areas (anterior cingulate gyri) and the basal ganglia. The severity of this syndrome may vary ranging from complete unresponsiveness to partial responsiveness during which the patient occasionally produces speech and intentional limb movements, though slowly and imperfectly. According to the clinical evaluation with the Coma Recovery Scale-Revised (CRS-R) **Patient 2** was not able to functionally manipulate objects even if he could still communicate with yes or no responses but not intelligible words. He was conscious although unable to exhibit motor and speech intentional initiation. ***Patient 3*** was diagnosed as a minimally conscious state plus (MCS+) according to the CRS-R. The disorder's origin was of vascular etiology; more precisely, the patient suffered from a severe subarachnoid hemorrhage following a rupture of the right middle cerebral artery. MCS is a disorder of consciousness in which minimal but definite behavioral evidence of self or environmental awareness is demonstrated by a standardized clinical assessment. MCS has been fully defined in 2002 to be differentiated from the Unresponsive Wakefulness Syndrome (UWS) (i.e., also known as vegetative state) in which only reflexive behavior can be recognized. Differently from LIS and Akinetic Mutism, this condition cannot be ascribed to a specific anatomical pattern of lesions but rather it may arise from a heterogenous spectrum of widespread cortical and/or subcortical lesions of different etiologies (vascular, metabolic, traumatic, and anoxic). In the MCS condition, patients show preserved arousal level and exhibit discernible but fluctuating signs of awareness. These signs may include high-level (MCS *plus*) behavioral responses (e.g. command following or intelligible verbalization) or low-level (MCS *minus*) non-reflexive responses (e.g. visual pursuit or localization of noxious stimulation). According to the clinical evaluation with CRS-r on the day of the experiment, Patient 3 was not able to communicate and produce any verbal output. However, she could understand the meaning of simple commands. Sometimes she could follow commands but with a fluctuating behavioral performance.

**Supplementary Table 5. Full model predictions on Patients.**

| **Patient ID** | **Day of experiment** | **EEG cumulative amplitude** | **EOG time to peak** | **EOG amplitude** | **Condition** | **Probability** | **Predicted** | **Correct** |
| --- | --- | --- | --- | --- | --- | --- | --- | --- |
| Patient 1 | 1 | 221,31 µV | 762,47 µV | 85,54 µV | intentional | 1 | intentional | TRUE |
| Patient 1 | 1 | 42,05 µV | 594,50 µV | 42,46 µV | spontaneous | 0.9 | intentional | FALSE |
| Patient 2 | 1 | 1,32 µV | 332,95 µV | 225,95 µV | intentional | 0.9 | intentional | TRUE |
| Patient 2 | 1 | 228,61 µV | 59,04 µV | 149,23 µV | spontaneous | 0.001 | spontaneous | TRUE |
| Patient 2 | 2 | -387,46 µV | 162,58 µV | 250,65 µV | intentional | 0.8 | intentional | TRUE |
| Patient 2 | 2 | -362,07 µV | 161,88 µV | 158,69 µV | spontaneous | 0.6 | spontaneous | TRUE |
| Patient 2 | 3 | -880,78 µV | 164,33 µV | 338,24 µV | intentional | 0.9 | intentional | TRUE |
| Patient 2 | 3 | 750,31 µV | 48,13 µV | 186,98 µV | spontaneous | 0.0001 | spontaneous | TRUE |
| Patient 3 | 1 | -1.078,10 µV | 359,90 µV | 614,18 µV | intentional | 0.9 | intentional | TRUE |
| Patient 3 | 1 | 539,00 µV | 94,48 µV | 750,06 µV | spontaneous | 0.18 | spontaneous | TRUE |
| Patient 3 | 2 | -2.866,16 µV | 63,05 µV | 546,05 µV | intentional | 0.9 | intentional | TRUE |
| Patient 3 | 2 | -1.256,64 µV | 275,45 µV | 753,54 µV | spontaneous | 0.9 | intentional | FALSE |
| Patient 3 | 3 | -1.506,07 µV | 220,06 µV | 576,25 µV | intentional | 0.9 | intentional | TRUE |
| Patient 3 | 3 | 2555,92 µV | 85,47 µV | 537,23 µV | spontaneous | 0.0000001 | spontaneous | TRUE |

**Software**

Programming Languages: Matlab 2015b; Python 3.10.6; R 4.2.1. Matlab libraries: EEGLAB (eeglab 14_0_0b). Python libraries: Mne 1.3.1; Numpy 1.23; Pandas 1.4; Matplotlib 3.5.3. R libraries: ggplot2 3.3.6; ez 4.4-0; dplyr 1.1.0; tidyr 1.2.0; nnet 7.3-17; pROC 1.18.0; caret 6.0-93; PRROC 1.3.1; pwrss 0.3.0.
